# Development of the interRAI Pressure Ulcer Risk Scale (PURS) for use in long-term care and home care settings

**DOI:** 10.1186/1471-2318-10-67

**Published:** 2010-09-20

**Authors:** Jeff Poss, Katharine M Murphy, M Gail Woodbury, Heather Orsted, Kimberly Stevenson, Gail Williams, Shirley MacAlpine, Nancy Curtin-Telegdi, John P Hirdes

**Affiliations:** 1 Department of Health Studies and Gerontology, University of Waterloo, Waterloo, ON, Canada; 2 Department of Primary Care Internal Medicine, Fletcher Allen Health Care, Burlington, VT, USA; 3 The Faculty of Health Sciences, The University of Western Ontario, London, ON, Canada; 4 Canadian Association of Wound Care, Toronto, ON, Canada; 5 Fairmount Home, Glenburnie, ON, Canada; 6 Ontario Ministry of Health and Long-Term Care, Toronto, ON, Canada; 7 Homewood Research Institute, Guelph, ON, Canada

## Abstract

**Background:**

In long-term care (LTC) homes in the province of Ontario, implementation of the Minimum Data Set (MDS) assessment and The Braden Scale for predicting pressure ulcer risk were occurring simultaneously. The purpose of this study was, using available data sources, to develop a bedside MDS-based scale to identify individuals under care at various levels of risk for developing pressure ulcers in order to facilitate targeting risk factors for prevention.

**Methods:**

Data for developing the interRAI Pressure Ulcer Risk Scale (interRAI PURS) were available from 2 Ontario sources: three LTC homes with 257 residents assessed during the same time frame with the MDS and Braden Scale for Predicting Pressure Sore Risk, and eighty-nine Ontario LTC homes with 12,896 residents with baseline/reassessment MDS data (median time 91 days), between 2005-2007. All assessments were done by trained clinical staff, and baseline assessments were restricted to those with no recorded pressure ulcer. MDS baseline/reassessment samples used in further testing included 13,062 patients of Ontario Complex Continuing Care Hospitals (CCC) and 73,183 Ontario long-stay home care (HC) clients.

**Results:**

A data-informed Braden Scale cross-walk scale using MDS items was devised from the 3-facility dataset, and tested in the larger longitudinal LTC homes data for its association with a future new pressure ulcer, giving a c-statistic of 0.676. Informed by this, LTC homes data along with evidence from the clinical literature was used to create an alternate-form 7-item additive scale, the interRAI PURS, with good distributional characteristics and c-statistic of 0.708. Testing of the scale in CCC and HC longitudinal data showed strong association with development of a new pressure ulcer.

**Conclusions:**

interRAI PURS differentiates risk of developing pressure ulcers among facility-based residents and home care recipients. As an output from an MDS assessment, it eliminates duplicated effort required for separate pressure ulcer risk scoring. Moreover, it can be done manually at the bedside during critical early days in an admission when the full MDS has yet to be completed. It can be calculated with established MDS instruments as well as with the newer interRAI suite instruments designed to follow persons across various care settings (interRAI Long-Term Care Facilities, interRAI Home Care, interRAI Palliative Care).

## Background

Pressure ulcers are an important and potentially preventable problem in LTC homes [1]. Estimates of the incidence of pressure ulcers vary among facilities from 6.5% [2] to 23.9% [3], despite the availability of validated tools [4] for evaluating an individual's risk of developing a pressure ulcer and clinical guidelines for prevention and care [5]. The key risk factors reported in the literature are impaired mobility, under-nutrition and low body mass index (BMI), poor physical health including factors affecting oxygenation and perfusion, advanced age, body temperature, friction and shear, skin moisture, pain, drugs, some kinds of medical devices, and impaired cognition and sensory perception [6-11]. Prevention is important for protecting or improving quality of life but is often costly and time consuming. Therefore, it must be carefully targeted based on risk factors for the outcome of interest [12]. As illustrated by the development and implementation of quality measures [13] for determining the quality of long-term care, pressure ulcer prevention has become a major focus of quality improvement initiatives across the US and Canada [1,14].

In 2007 the Ontario Ministry of Health and Long-Term Care sponsored the implementation of a Pressure Ulcer Awareness and Prevention Program (PUAP Program) by the Canadian Association of Wound Care (CAWC) in Ontario LTC homes. The basis of the PUAP Program is risk assessment and intervention using the Braden Scale[4]. Within the program LTC staff complete the Braden Scale at admission, then every week for four weeks and quarterly thereafter [5], or more frequently if a change in health status occurs. Resources and plan of care are based on a resident's risk level and individual sub-scale scores. Completing both the mandated MDS [15] and the Braden Scale presented LTC home staff with considerable assessment and paperwork burden, often perceived as duplicated effort. This perception may place the PUAP at potential risk of facility non-adherence to protocols, and residents at risk of pressure ulcer development.

Few studies have been published that examine the Braden Scale and the MDS together. A chart review of 8 US homes in which MDS and Braden Scale data were available compared the Braden Scale and the MDS Resident Assessment Protocol (RAP), concluding that there was good potential for MDS information to take the place of the Braden Scale [16]. An examination of MDS items and Braden Scale domains indicated that some Braden Scale domains are not well-operationalized in the MDS (nutritional status and friction/shear), and some that are available (sensory perception items) are not used in the pressure ulcer RAP [17]. Braden Scale data were collected along with MDS data in the National Pressure Ulcer Long-Term Care Study, with MDS items used primarily as descriptor variables [18].

The purpose of this study was to examine the potential of the MDS assessment, mandated for use in Ontario LTC homes, to inform the risk for pressure ulcers. Specifically, the study sought to simulate Braden Scale domains with MDS items, using clinical expert input, and use this as a starting point to develop a bedside MDS-based scale to identify LTC home residents at various levels of risk for developing pressure ulcers, in order to facilitate targeting of risk factors for prevention.

## Methods

### Instruments

The Resident Assessment Instrument (RAI), developed in response to 1987 US Nursing Home Reform laws, aims to provide a comprehensive assessment of nursing home residents [15]. The cornerstone of the RAI is the MDS, a uniform, standardized, computerized tool for assessing multiple domains of a person's physical, social and psychological health and function, including skin integrity, number and stage of pressure ulcer(s) and typical risk factors for pressure ulcer development noted in the literature [2]. The MDS assessment tool has received extensive reliability and validity testing [19]. The RAI User's Manual directs nursing home staff to complete a full MDS evaluation of each resident by admission day 14, annually, and upon major change in functioning, with a subset of items completed quarterly [20]. The RAI also includes eighteen RAPs that are triggered by specific MDS item responses. RAPs provide clinicians with support for conducting a more in-depth assessment for prevention and care planning of potential or actual problems, such as Pressure Ulcers [21]. These RAPs have recently been updated as part of an international effort to combine new knowledge gained from the use of the RAI around the globe with current best practice guidelines in a variety of areas including pressure ulcers [22].

RAI Version 2.0 has been utilized in the US since 1996, and Ontario LTC homes began implementing it as the standard of care in 2005. It has been translated into 18 languages and is used internationally for care planning, facility management, needs assessment, policy development, quality improvement and benchmarking, reimbursement, research, or service eligibility, making it the most widely used comprehensive assessment instrument world-wide. An updated version has recently been completed by the interRAI research network (http://www.interrai.org) [23].

The Braden Scale for Predicting Pressure Sore Risk [4] is likely the most widely used tool in facility-wide pressure ulcer prevention programs in North America. The Braden Scale is a summary measure comprised of six sub-scales for measuring an individual's functional determinants of pressure and tolerance of tissues to withstand pressure. Each of the six subscales (activity, mobility, sensory perception, nutrition, moisture, and friction and shear) is scored 1 (least favourable) to 4 (most favourable), with the exception of friction and shear which is scored from 1 to 3. Scores can range from 6 to 23; lower scores are associated with higher risk for developing pressure ulcers.

Risk assessment tools, including the Braden Scale, have been criticized for their generally weak properties [24], although among tools with published findings, a recent review article found the Braden Scale to perform better than others [25]. The Braden Scale domains of nutrition and sensory perception have been shown to have poor reliability [26]. It is notable that there is a lack of good evidence that the use of any pressure ulcer risk scale actually reduces pressure ulcers in clinical practice [27].

### Study Overview

The goal of this work was to investigate the possibility of an MDS-informed pressure ulcer risk scale that could eliminate duplicated assessment burden. Being the product of three parties (wound care education, government health care administration, health research), the investigation was often formative in nature, characterized by several phases:

1) Identification of existing LTC homes (a small convenience sample) where both Braden Scale and MDS data were collected concurrently, and using wound care expertise along with evidence from these data to map the Braden Scale using MDS items,

2) Testing the ability of this mapped Braden Scale to predict a new pressure ulcer among those without a pressure ulcer, in a much larger sample,

3) Consideration of other MDS items that might improve predictive performance.

### Setting and Subjects

Beginning in 2005, Ontario LTC homes volunteered to implement the MDS assessment as standard practice, with groups of homes receiving training and support in a phased roll-out to all homes in the province. By the fall of 2007, 89 of the province's more than 600 homes had submitted MDS data to the national reporting system at the Canadian Institute for Health Information (CIHI). Data included an admission or baseline MDS assessment of each resident having a stay of 14 days or longer, and subsequent reassessments every 90 days (or earlier in the case of a clinically significant change in health or functional status). A total of 72,602 assessments, with a resident identifier allowing longitudinal linkage but keeping identity anonymous, were available for analysis. From these data, an analytic dataset was created of all unique individuals using their baseline assessment/reassessment pair, for a total of 14,083, of which 91.6% (n = 12,896) had no recorded pressure ulcer at the baseline assessment. The median time between assessments was 91 days.

Three LTC homes were identified as collecting Braden Scale scores and MDS assessment data. The Braden Scale information included the 6 sub-scores and were captured electronically and linked through a non-real-world identifier to extracted MDS data, yielding a dataset of 257 cases. The median time difference between MDS and Braden Scale assessments was 9 days.

In addition to the derivation data from Ontario LTC homes, data from other settings were drawn from holdings available to interRAI researchers, in order to test pressure ulcer prediction. Ontario CCC hospital MDS assessments were drawn from 2002 to 2007. This population differs from Ontario LTC home residents, primarily in that they tend to have shorter stays, require post-acute care, are more clinically unstable, and are more likely to receive rehabilitation services. A baseline assessment/reassessment sample was constructed using the same methodology as for LTC homes, resulting in a sample of 17,956, of which 72.7% (n = 13,062) had no recorded pressure ulcer at baseline. Also of interest are long-stay home care clients assessed with the RAI-Home Care (RAI-HC) [28] by Ontario home care case managers as part of routine clinical practice. A sample of community-assessed clients was drawn using a similar baseline assessment/reassessment approach, resulting in 76,068 clients, of which 96.2% (n = 73,183) had no recorded pressure ulcer at baseline. A subset of these home care clients assessed in acute care hospital and identified for long-term care placement was identified for comparison. In addition, a cross-sectional research sample assessed using the interRAI Palliative Care instrument [29] was available (n = 988), to explore the characteristics of community-dwelling palliative clients and pressure ulcers in Ontario.

### MDS Assessment of Pressure Ulcers

Assessors trained in the MDS record the presence, stage, and number of pressure ulcers in the last 7 days. Ulcers were staged by the following criteria: (1) persistent area of redness (without a break in the skin) that does not disappear when pressure is relieved, (2) a partial thickness loss of skin layers that presents clinically as an abrasion, blister or shallow crater, (3) a full thickness of skin is lost, exposing subcutaneous tissues (presents as a deep crater with or without undermining adjacent tissue), and (4) a full thickness of skin and subcutaneous tissue is lost, exposing muscle or bone [20].

### Analysis

For the matched Braden Scale-MDS dataset (n = 257), Spearman rank correlations between each of the 6 Braden sub-scores and candidate MDS items and scales (identified in a series of telephone meetings among the three parties) were examined in order to inform a possible cross-walk of the Braden Scale to the MDS. Subsequently, various constructions of a Braden Scale cross-walk were considered, informed both by clinical insight/face validity as well as strength of correlation. The cross-walk scale versions were constructed to mimic the Braden Scale both in sub-scale and total score ranges. Insufficient pressure ulcer incidence data were available for this small dataset, so these cross-walk algorithms were applied to the larger baseline/reassessment data in LTC homes to examine performance in predicting new pressure ulcers among those without pressure ulcer at baseline. The cross-walk versions were evaluated using logistic regression, predicting a new pressure ulcer at the next quarterly assessment, with the C-statistic (area under the receiver operator characteristic curve) as the main evaluation criterion. The interpretation of the C-statistic of 0.7, for example, in this case is that a randomly chosen individual who goes on to develop a pressure ulcer is likely to have a higher scale score than a randomly chosen individual who does not develop a pressure ulcer 70% of the time. Values of 0.5 reflect no better than chance alone, and higher numbers indicate better diagnostic prediction, with a value of 1.0 being perfectly accurate [30]. The value of the C-statistic will be used to differentiate relative performance of differently constructed scales within the same data.

Keeping the Braden Scale findings in mind, a series of exploratory analyses was subsequently done using the larger Ontario LTC homes baseline/reassessment dataset alone, to explore if other constructions of MDS items could improve on the Braden Scale cross-walk in predicting new pressure ulcers. Here the methods included both multivariable logistic regression (to test independent effects) and decision tree modelling (to try to discover potential interaction effects), using as the dependent variable new pressure ulcer at reassessment among those without a pressure ulcer at baseline. Evidence from the literature and clinical expertise contributed to the refinement of these models, with the goal of a predictive scale that could be calculated using an existing MDS assessment, but also could be readily done at the bedside prior to completion of the full set of MDS items by admission day 14, or prior to a scheduled MDS reassessment. Logistic regression of new pressure ulcer was used to determine those MDS items statistically significant (at p = 0.05 or below) in a multivariable, parsimonious model. These items were applied in an interactive decision tree model (with potential splits based on chi-square values for a new pressure ulcer and the candidate splitting variable) as a final step to see if alternative treatment of them might improve predictive performance.

The final scale was replicated in other interRAI instruments, here the home care and palliative care tools to examine how it was associated with incidence of pressure ulcers in other at-risk populations.

SAS and SAS Enterprise Miner Tree Desktop version 9.1.3 were used for all analyses.

Data sharing agreements allowed for the transfer of anonymized data from both CIHI and the Ontario Ministry of Health and Long-Term Care to the University of Waterloo, and ethics approval for analysis of anonymized data was in place from the Office of Research Ethics, University of Waterloo.

## Results

Characteristics of residents assessed with the Braden Scale-MDS and baseline/reassessment MDS are presented as Table 1. Demographic information was unavailable for the Braden Scale sample, but the larger sample presents residents who are elderly and predominately female. In both samples more than half had substantial problems in cognition and functioning in Activities of Daily Living (ADLs).

**Table 1:** Characteristics of Derivation Samples from Ontario Long-Term Care Homes

Resident Characteristic	Braden Scale Sample (n = 257)	Assessment/Reassessment SampleM (n = 14,083)
Age		
mean (std dev)	unavailable	82.2 (10.2)
< 65		6.3%
65-74		10.3%
75-84		37.0%
85+		46.5%

Female	unavailable	69.2%

Median day of stay at assessment	451	475

Pressure ulcer *(last 7 days)*		
none	93.4%	91.6%
stage 1	1.2%	3.3%
stage 2 or higher	5.4%	5.1%

Moderate cognitive impairment or greater (CPS* 3+)	54.9%	58.8%

Extensive assistance in ADL or greater (ADL hierarchy** 3+)	68.1%	74.7%

CHESS*** 2+	24.1%	17.2%

Diagnoses		
- Diabetes	18.7%	21.8%
- Congestive heart failure	13.3%	11.8%
- Alzheimer's/related dementia	45.1%	55.6%
- COPD	21.8%	12.9%
- Stroke	32.3%	20.7%

Braden Scale Total Score		
17-23	59.5%	
15-16 (low)	9.7%	
13-14 (moderate)	13.6%	
6-12 (high)	17.1%	

Incidence of pressure ulcer	--	3.9%

* CPS: Cognitive Performance Scale, possible scores 0 to 6 with higher scores indicating more severe cognitive impairment, values of 3 or greater equate to an MMSE score of approximately 15 or less

** ADL Hierarchy: Activities of Daily Living Hierarchy Scale, possible scores 0 to 6 with higher scores indicating more dependence in ADL, values of 3 or greater indicate extensive assistance required

*** CHESS: Changes in Health, End-stage disease and Sign and Symptoms of medical problems, possible scores of 0 to 5 with higher scores indicating more unstable health

Bivariate associations with the six Braden Scale component scores are presented in Table 2. Some of the candidate items from the MDS (notably ADLs like transfer, bed mobility, walking) are highly correlated with multiple Braden Scale domains. Of the six Braden Scale domains, activity, mobility, and friction/shear have the strongest candidate items in the MDS, sensory perception and moisture are slightly lower, and nutrition lower yet. Note that the negative correlations reflect the different forms of coding between Braden Scale (higher numbers, less impairment) and MDS items (higher numbers, more impairment).

**Table 2:** Association of MDS 2.0 items with Braden Scale Subscales in Ontario Long-Term Care Homes

Braden Scale Domain	Candidate items from MDS 2.0 (item code)	Correlation coefficient*
Sensory Perception	cognitive skills for decision making (B4)	-0.67
	transfer (G1ba)	-0.62
	making self understood (C4)	-0.50

Moisture	bowel incontinence (H1a)	-0.68
	bladder incontinence (H1b)	-0.54
	Personal hygiene (G1ja)	-0.52

Activity	walk in room (G1ca)	-0.85
	bed mobility (G1aa)	-0.80
	wheelchair use (G5d)	-0.77

Mobility	walk in room (G1ca)	-0.81
	transfer (G1ba)	-0.81
	bed mobility (G1aa)	-0.77

Nutrition	leaves food uneaten (K4c)	-0.48
	weight (K2b)	0.41
	body mass index (uses K2a, K2b)	0.38

Friction/shear	transfer (G1ba)	-0.75
	bed mobility (G1aa)	-0.72
	walk in room (G1ca)	-0.70

*Spearman Rank Correlation (all significant at p < .0001)

Using the best candidate single items available in each of the six domains to give weights equivalent to the Braden Scale scoring, a six-item cross-walk was constructed using the following MDS items: cognitive skills for daily decision making, bowel incontinence, walk in room, transfer, leaves food uneaten, and bed mobility. While suitable for informing a Braden cross-walk, it resulted in cell sizes that were too small for validation (i.e., prediction of a new pressure ulcer) in the sample of only 257.

**Figure 1:**
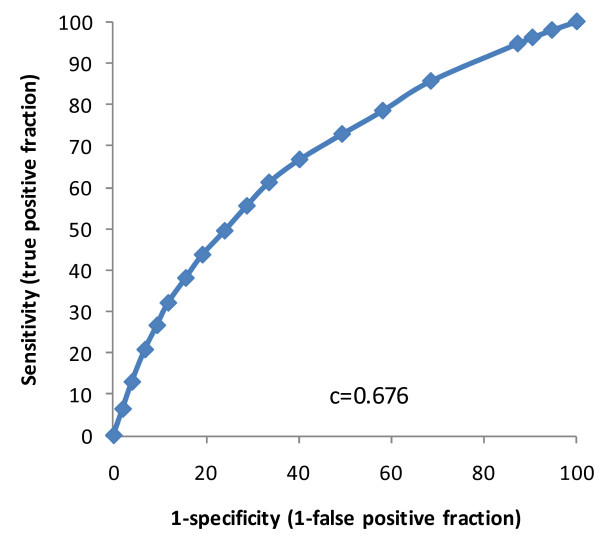
**Receiver Operator Curve, Braden Scale Cross-walk, LTC Homes**.

Shifting to the larger baseline/reassessment dataset (n = 12,896), the Braden Scale cross-walk total score was tested for its predictive ability, with pressure ulcer incidence ranging from about 9% down to about 1% across its scoring range from 6 (high risk) to 23 (low risk), respectively, and a c-statistic in the logistic regression model of 0.676. The receiver operator characteristic (ROC) curve is provided as Figure 1. The six component items were also tested in multivariable logistic regression models to examine if they all contributed in the prediction of a new pressure ulcer at reassessment. Of the six items, cognitive skills for decision making and transfer were not significantly associated with the dependent variable when adjusting for the other four. For reasons of compatibility with the newer generation interRAI instruments, weight loss was substituted for leaves food uneaten; while the two items were only weakly correlated with one another, weight loss had a much stronger association with a new pressure ulcer, and it was consistent with the spirit of the Braden Scale nutrition domain.

Additional candidate items were then considered based on the literature and clinical expert input, among them indwelling catheter, urinary incontinence, end-stage disease, BMI, history of pressure ulcers, daily pain, and shortness of breath. Decision tree modelling did not contribute insight that was helpful. However, several interactions among candidate items were tested in the logistic models, but they did not achieve statistical significance at the.05 level. The most parsimonious model used the following independent variables: bowel incontinence, walk in room, bed mobility, weight loss, history of resolved pressure ulcers, shortness of breath, and daily pain. All were represented as dichotomous values. Item prevalence in the baseline LTC residents as well as odds ratios for new pressure ulcer at reassessment are presented in Table 3.

**Table 3:** Prevalence and odds ratios for interRAI PURS scale items with incidence of pressure ulcer in Ontario Long-Term Care Homes

interRAI PURS items	Prevalence	New Pressure Ulcer: Odds Ratio (95% confidence)	
		Bivariate	Multi-variable Model
Impaired in bed mobility (G1aa = 3,4 or 8)	44.1%	3.05 (2.52, 3.69)	1.66 (1.30, 2.13)

Impaired in walking (G1ca = 3,4 or 8)	50.7%	3.10 (2.54, 3.79)	1.63 (1.26, 2.12)

Bowel incontinence (H1a = 2,3 or 4)	51.1%	2.80 (2.30, 3.42)	1.78 (1.42, 2.24)

Weight loss (K3a = 1)	19.7%	1.43 (1.16, 1.76)	1.55 (1.25, 1.91)

History of resolved pressure ulcers (M3 = 1)	7.8%	3.53 (2.74, 4.53)	2.47 (1.91, 3.19)

Daily pain (J2a = 2)	19.3%	1.45 (1.18, 1.78)	1.41 (1.14, 1.75)

Shortness of breath (J1l = 1)	6.3%	1.45 (1.05, 2.00)	1.54 (1.11, 2.14)

**Figure 2:**
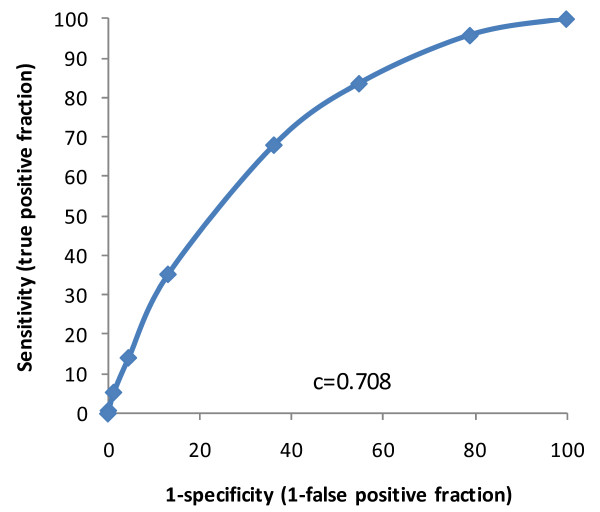
**Receiver Operator Curve, interRAI PURS Scale, LTC Homes**.

**Figure 3:**
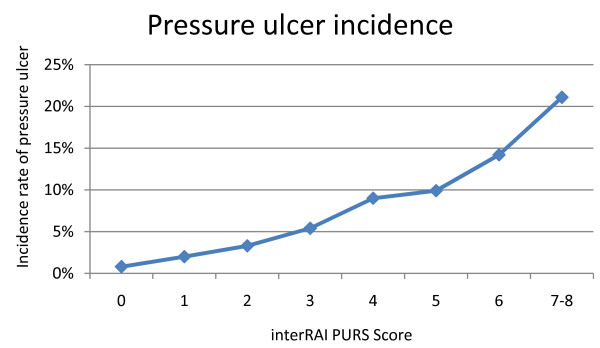
**Pressure Ulcer Incidence by interRAI PURS Score, LTC Homes**.

Of the seven items, history of resolved pressure ulcer was the strongest predictor of new pressure ulcers at follow-up in the multivariable model, with an odds ratio of 2.5. Other items had odds ratios between 1.4 and 1.8. An additive score was then constructed with history of resolved pressure ulcer counting for two points if present, and the other six items counting one if present, resulting in a scale of 0 to 8 (higher numbers representing higher risk of a new pressure ulcer at reassessment). Values of 8 were very rare (2 cases only), and were grouped with the 7's. This additive scale achieved a c-statistic of 0.708 in the model predicting new pressure ulcers. Results are shown in Table 4, Figure 2 and Figure 3, with increasing incidence rates and odds ratios by increasing scale value. The new scale is named the interRAI Pressure Ulcer Risk Scale (interRAI PURS).

**Table 4:** interRAI PURS Performance Among Ontario Long-Term Care Home Residents with No Pressure Ulcer at Baseline

interRAI PURS Score	Proportion of residents at baseline	Pressure ulcer rate at reassessment	Odds ratio, 95% confidence
0	18.9%	0.8%	Reference

1	22.3%	2.0%	2.53 (1.53, 4.16)

2	17.9%	3.3%	4.21 (2.59, 6.83)

3	24.0%	5.4%	7.11 (4.50, 11.23)

4	10.1%	9.0%	12.28 (7.65, 19.72)

5	4.7%	9.9%	13.62 (8.01, 23.14)

6	1.8%	14.2%	20.53 (11.09, 38.01)

7-8	0.3%	21.1%	33.11 (10.14, 108.13)

The seven independent variables in interRAI PURS were also used in modeling new pressure ulcer using an interactive decision tree tool. Similar associations to the regression results were found, and generally parallel structures were supported among candidate tree models. Predictive performance was slightly weaker than the additive model, with c-statistic values around 0.690, and when considering the additional complexity of scoring an algorithm or tree at the bedside, this approach was not pursued.

Applying the interRAI PURS score to Ontario CCC patients without a pressure ulcer at their initial assessment (n = 13,062), distribution of the scale was skewed more towards the high risk end (only 20% scored 0 or 1, compared to over 41% among LTC residents). This was consistent with an overall higher pressure ulcer incidence rate of 9.7% in CCC compared to 3.9% in LTC. The scale values showed consistently higher pressure ulcer incidence with higher scores (4.4% at scale value 0, to 23.7% at scale value 7). The c-statistic in this sample was 0.607.

In long-stay home care clients, interRAI PURS is skewed more towards lower risk, with nearly three quarters of clients scoring 0 or 1. Risk of pressure ulcer incidence was in fact much lower in this population compared to either LTC or CCC at about 2.6%. The scale was able to clearly and consistently differentiate risk, with incidence rates ranging from 1.6% among those scoring 0, to 13.1% for those scoring 6 or greater. Here, the c-statistic for interRAI PURS was 0.629.

Standardized MDS assessment information supports an examination of how the interRAI PURS scale distributes in multiple settings, and this is presented as Table 5. As one moves from community-dwelling home care clients, LTC facility residents, community-based palliative care clients, hospital-assessed LTC-bound patients to CCC patients there is a clear shift in interRAI PURS distributions towards higher risk scores as well as higher incidence. Pressure ulcer incidence information is not available for palliative care and hospital-assessed individuals, but this summary provides an indication of concurrent validity whereby prevalence of pressure ulcers in various settings is associated with risk as measured with the interRAI PURS scale. It should be noted that this table presents distribution of the interRAI PURS across all assessed individuals, including those with pressure ulcers, as opposed to the baseline/reassessment samples that included only individuals without pressure ulcers at the baseline assessment.

**Table 5:** interRAI PURS Distribution and Pressure Ulcer Prevalence in Various Settings, Ontario 2002-7

interRAI PURS Score	Home Care	Long-Term Care*	Community Palliative Care	Acute Hospital (Alternate Level of Care)	Complex Continuing Care
0	29.3%	19.0%	14.3%	18.6%	6.3%

1	40.8%	19.9%	30.9%	26.7%	14.1%

2	19.6%	17.0%	29.1%	20.1%	17.6%

3	6.9%	25.1%	14.9%	17.1%	23.7%

4	2.5%	10.7%	7.0%	10.4%	21.9%

5	0.7%	6.0%	1.9%	4.7%	11.5%

6	0.2%	1.6%	1.5%	1.8%	3.8%

7-8	0.1%	0.6%	0.5%	0.7%	1.1%

Proportion with any pressure ulcer	3.8%	8.0%	9.8%	15.5%	27.3%

*cross-sectional sample, differs from baseline/reassessment sample used in scale derivation

## Discussion

interRAI PURS provides an estimate of graded risk for developing a pressure ulcer among individuals who do not present with a pressure ulcer at the time of assessment. It can be calculated using a completed MDS assessment, presumably as a computer-generated output similar to other MDS-based scales and clinical assessment protocols. It has a simple 7-item additive structure which lends itself to manual bedside scoring during early admission periods prior to completion of the full set of MDS items, or prior to a scheduled MDS reassessment. Since it uses items drawn directly from the MDS, assessor training skills and resources already present in that home or organization can be drawn upon, minimizing any duplication often required to support a separate pressure ulcer clinical screening program. interRAI PURS can be calculated using interRAI instruments in various settings, as well as for the new integrated suite of instruments, including the long-term care facilities (interRAI LTCF), home care (interRAI HC), and palliative care (interRAI PC) in which good psychometric properties have been demonstrated [31].

Of note is the property that the scale distributes roughly one-fifth of the assessed residents into each of the lowest four scale values among LTC residents, with progressively fewer in each of the higher risk scores. This makes it possible to identify increasingly rare individuals at the highest level of risk. Identification of such individuals may add clinical value, as pressure ulcer risk is associated with high need for care due to functional deficits [32].

The ability of interRAI PURS to predict individuals at higher risk of a new pressure ulcer could not be directly compared to established instruments such as the Braden Scale from data available in this study, since the concurrent Braden and MDS observations were too few to establish stable estimates for pressure ulcer incidence. However, by constructing a cross-walk of the Braden Scale using evidence from the small matched dataset, a much larger sample of MDS assessments could be employed. Clinical expert input was used to guide the choice of domain areas and items, and hence the process aimed to strike a balance between evidence available in the data and in the clinical literature. While the Braden cross-walk using MDS items was informative, it was not pursued due to the failure of two of the six Braden domains to predict new pressure ulcers in the MDS data, along with the superior performance of interRAI PURS.

Given that the starting point of this work was the Braden scale, it is notable that of its six sub-scales, many have parallels in interRAI PURS: mobility (walking); nutrition (weight loss); friction/shear (bed mobility). As well, shortness of breath can be related to the Braden Scale activity domain. The sensory perception sub-scale of the Braden Scale does not have an analogous item in interRAI PURS. Also, two items in interRAI PURS do not match to an analogous domain in the Braden: daily pain and history of resolved pressure ulcer, although both are described in the literature as risk factors for pressure ulcer development [33,34].

Several considerations need to be observed from a clinical perspective with any summary scale such as the interRAI PURS. The quality of contributing items can be dependent on the skill of the assessor, and consistent training and monitoring of MDS assessment skills are important. The administration of interRAI PURS as close to the admission as possible, along with reassessment conditioned on baseline risk and clinical change (not merely when the next MDS quarterly assessment is due) are also central to an effective pressure ulcer program. RAPs provide clinicians with a structured, problem and prevention-oriented approach to care planning for potential or actual problems, such as pressure ulcers, and may be used along with summary scales such as interRAI PURS to guide care planning and targeting of pressure ulcer program activities. Longitudinal change in interRAI PURS score should also be tracked to signal changes in care plans. interRAI PURS, like any automated scale, should be used to support but not replace clinical judgment.

interRAI PURS may be used clinically both as an automated output from a computer-entered MDS assessment, as well as from a manually completed bedside form. Next steps will need to evaluate how interRAI PURS performs as a clinical tool, considering both usability and clinical outcomes at the bedside. In Ontario, a collaborative pressure ulcer awareness program in LTC homes is underway that will be evaluating both interRAI PURS and the process of its implementation. While the interRAI PURS has been shown to identify individuals more likely to develop a pressure ulcer in the future, it cannot be assumed that its use as a clinical indicator to guide practice will reduce the incidence of pressure ulcers. In fact, there is a lack of high quality evidence of clinical effectiveness for any risk scale of this kind [27]. Randomized controlled trial quality studies would be required to show that assessing pressure ulcer risk is more than a burden to care staff. However, with risk assessment firmly established in clinical practice, the interRAI PURS can reduce duplication in this assessment burden.

Of note in this study is the low reported prevalence of pressure ulcers in the long-term care sample, at 8.4%. While this falls near the lower boundary of reported rates, it may underreport some residents with pressure ulcers, in particular Stage 1 ulcers. This is not entirely surprising as detection of Stage 1 types can be problematic [35]. In addition to basic issues of detection and recording, the 3-month interval of the MDS data will tend to under-report new pressure ulcers, as some will occur and subsequently heal before the new assessment look-back window, and risk factors may also change during this time. This work uses secondary data not explicitly collected for this purpose, and while data quality has generally been found to be good in jurisdictions like Ontario where MDS implementation was planned and supported [19], there is of course some degree of imprecision in measurement of both pressure ulcer and risk factors. However, these errors tend to attenuate associations in the data, supporting the robustness of results found. There is likely a bias in the data towards under-reporting the least important wounds (pressure ulcers quickly healed, minor stage 1 pressure ulcers); whether these types of individuals have distinct risk factors is unknown, but it is likely that measured risk factors operate in a similar fashion to those experiencing more severe pressure ulcers.

## Conclusions

This study provides a new application for MDS data to support interventions aimed at targeting care for those at risk of developing pressure ulcers. The reduction of assessment burden achieved by harmonizing the risk assessment with the MDS and the ability to derive this scale in multiple service settings make interRAI PURS an attractive scale for system-wide approaches to pressure ulcer reduction strategies.

## Competing interests

The authors declare that they have no competing interests.

## Authors' contributions

All authors participated in the conception and planning of the study. JP led the data management and analysis with valuable assistance in securing data from SM and her team at the Ontario Ministry of Health and Long-Term Care. All authors contributed to the drafting and revisions of the manuscript. All authors have read and approved the final manuscript.

## Pre-publication history

The pre-publication history for this paper can be accessed here:

http://www.biomedcentral.com/1471-2318/10/67/prepub
